# AP-TSS: A New Method for the Analysis of RNA Expression from Particular and Challenging Transcription Start Sites

**DOI:** 10.3390/biom10060827

**Published:** 2020-05-28

**Authors:** Gabriel Le Berre, Virginie Hossard, Jean-Francois Riou, Anne-Laure Guieysse-Peugeot

**Affiliations:** Structure et Instabilité des Génomes, Muséum National d’Histoire Naturelle, CNRS UMR 7196, INSERM U1154, 43 rue Cuvier, F-75005 Paris, France; gabriel.leberre@uclouvain.be (G.L.B.); virginie.hossard@mnhn.fr (V.H.); jean-francois.riou@mnhn.fr (J.-F.R.)

**Keywords:** alternative TSS, alternative promoter, TERRA, transcript isoform, transcript quantification, Alu

## Abstract

Alternative promoter usage involved in the regulation of transcription, splicing, and translation contributes to proteome diversity and is involved in a large number of diseases, in particular, cancer. Epigenetic mechanisms and cis regulatory elements are involved in alternative promoter activity. Multiple transcript isoforms can be produced from a gene, due to the initiation of transcription at different transcription start sites (TSS). These transcripts may not have regions that allow discrimination during RT-qPCR, making quantification technically challenging. This study presents a general method for the relative quantification of a transcript synthesized from a particular TSS that we called AP-TSS (analysis of particular TSS). AP-TSS is based on the specific elongation of the cDNA of interest, followed by its quantification by qPCR. As proof of principle, AP-TSS was applied to two non-coding RNA: telomeric repeat-containing RNAs (TERRA) from a particular subtelomeric TSS, and Alu transcripts. The treatment of cells with a DNA methylation inhibitor was associated with a global increase of the total TERRA level, but the TERRA expression from the TSS of interest did not change in HT1080 cells, and only modestly increased in HeLa cells. This result suggests that TERRA upregulation induced by global demethylation of the genome is mainly due to activation from sites other than this particular TSS. For Alu RNA, the signal obtained by AP-TSS is specific for the RNA Polymerase III-dependent Alu transcript. In summary, our method provides a tool to study regulation of gene expression from a given transcription start site, in different conditions that could be applied to many genes. In particular, AP-TSS can be used to investigate the epigenetic regulation of alternative TSS usage that is of importance for the development of epigenetic-targeted therapies.

## 1. Introduction

Regulation of gene expression occurs at a number of levels. The use of alternative promoters can regulate gene expression levels and can increase protein diversity. A large fraction of human genes possess multiple alternative promoters that reportedly control mRNA splicing, transcription, and translation [1,2,3]. Alternative promoter use has been shown to be involved in a large number of diseases. For example, genes related to cancer displayed a higher than average number of alternative promoters [2]. Moreover, in colorectal cancer cells, tumor-specific usage of particular promoters has been found for some genes [4]. Epigenetic mechanisms [5,6] and cis regulatory elements [6,7] are involved in alternative promoter activity. The study of promoter use requires a specific quantification of transcripts from each transcription start site (TSS). 

**Figure 1:**
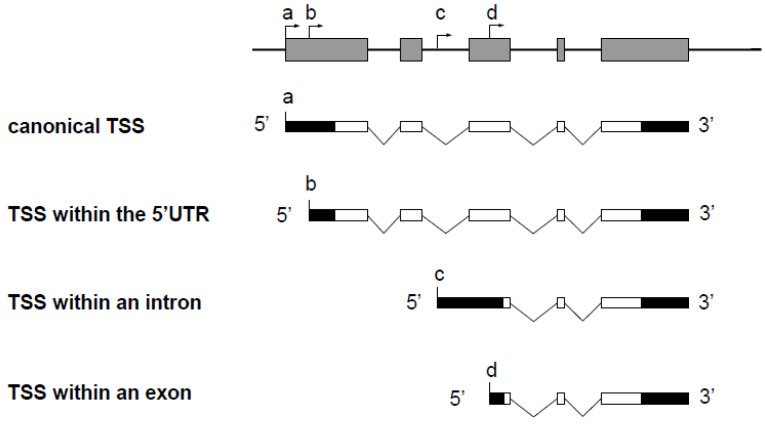
Schematic representation of a spliced gene and transcript isoforms synthesized from multiple TSSs. Exons and introns of the gene are shown as grey boxes and black lines, respectively. Untranslated and translated regions of the transcript isoforms are indicated as black boxes and white boxes, respectively. The TSS of the canonical transcript is located at the position marked (a). Transcripts from a TSS located within an intron (c) possess a unique sequence due to an alternative first exon. In contrast, transcripts from a TSS located within the 5′ UTR (b) or within an exon (d) do not possess unique sequence compared to the canonical transcript.

The use of alternative gene promoters generates different transcript isoforms. When the alternative promoter is located within an intron of the gene (Figure 1, TSS c), the transcript isoform possesses a unique sequence corresponding to the alternative first exon. Consequently, this isoform can be quantified by classical RT-qPCR with specific primers, which is the most sensitive and accurate method to study gene expression. In three situations, however, the transcripts from an alternative TSS do not contain unique sequences that enable discrimination from other transcript isoforms by classical RT-PCR (Figure 1). 

**Figure 2:**
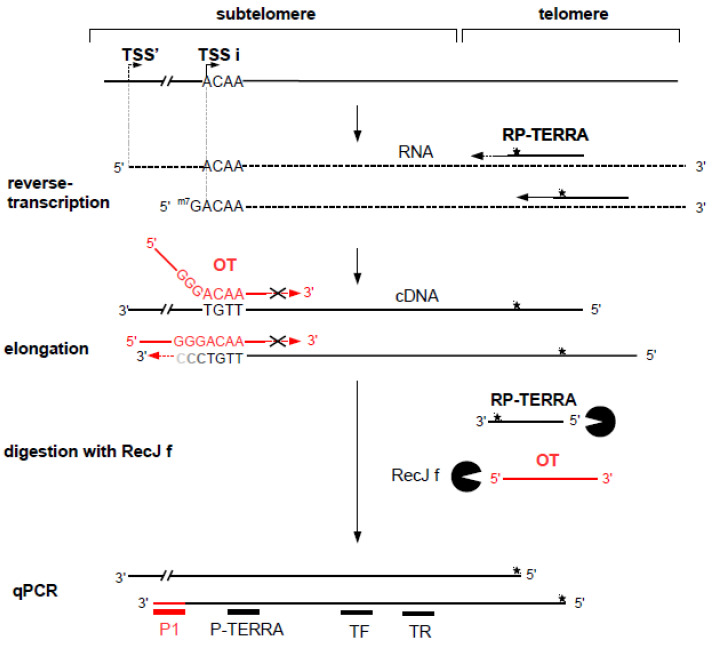
Schematic representation of the method for TERRA quantification from a single transcription start site (TSS) by analysis of particular TSS (AP-TSS). TERRA transcripts are produced from multiple TSSs located on subtelomeres. Two TSSs are illustrated here: the TSS of interest (TSSi) and a putative TSS located upstream (TSS’). TERRA (dashed line) is reverse transcribed using primer RP-TERRA complementary to the telomeric G-rich strand. As this primer can bind to sites throughout the telomeric repeat sequence, the 5′ ends of the cDNAs produced are of various lengths. Phosphorothioate bonds in the 3′-terminal part of RP-TERRA are represented with a black star. The produced cDNAs have subtelomeric sequences of variable length, depending on the TSS of origin. Template oligonucleotide (OT) (red) hybridizes with the 3′ end of the cDNA from the TSSi and serves as template for specific elongation of the cDNA. OT is blocked at its 3′ end by dideoxycytidines to prevent elongation. Oligonucleotides RP-TERRA and OT are digested by the 5′ exonuclease RecJf; the phosphorothioate bonds in the 3′-terminal part of RP-TERRA protect the cDNAs from digestion. Elongated cDNAs are quantified by qPCR using P1 and P-TERRA primers. TR and TF primers allow quantification of total TERRA from subtelomeres Xq, Yq, 15q, and 9p.

In the first case, the alternative TSS is located within the 5′ untranslated region (5′ UTR) of the gene, and the alternative transcript possesses a 5′ UTR different from that of the canonical transcript (Figure 1, TSS b), which can influence translation efficiency [8,9]. In the second case, the alternative TSS is located in a coding exon (Figure 1, TSS d), and the alternative transcript can be translated into an amino-terminal truncated protein that may have a function which is different from the full-length protein. For example, the *netrin-1* gene encodes a secreted protein involved in axon guidance. An alternative TSS located in the exon 2 leads to the production of a truncated protein that remains in the nucleus and that is proto-oncogenic [10]. In the third case, a transcript from a TSS located downstream from another TSS in an unspliced gene does not contain unique sequence. Notably, such a profile is also found in the non-coding telomeric repeat-containing RNA (TERRA) (Figure 2) [11]. In such cases, transcript isoforms are devoid of unique sequences and cannot be discriminated from the other isoforms by RT-qPCR. The unique feature, compared with the other transcript isoforms encoded by the same gene, is the length of the 5′ end, which depends on alternative TSS localization. The methods known as 5′ RACE (for Rapid Amplification of cDNA Ends) [12,13,14,15] and CAGE (for Cap Analysis of Gene Expression) [16] enable the identification of a TSS by addition of an adaptor at the 3′ end of the cDNA followed by amplification using a primer complementary to the adapter sequence. Sequencing using short-read counts enables estimation of the number of transcripts from a TSS. However, the efficiency of the adaptor addition step (by ligation or template switching) is not reliable enough to provide accurate quantification. Moreover, the adaptor is added at the 3′ end of all cDNAs present in the sample, which does not allow specific PCR amplification of the cDNA of interest. Finally, these methods are expensive and time consuming.

In this work, we describe an alternative method for relative quantification by the RT-qPCR of transcripts from a particular TSS, in which the addition of the tag sequence is not ligation-mediated and is specific for the cDNA of interest. We have applied this method to the non-coding telomeric repeat-containing RNA TERRA and to the Alu transcripts. 

TERRAs are heterogeneous long non-coding RNAs transcribed from subtelomeric TSSs towards chromosome ends. These RNAs contain both a subtelomeric and a telomeric tract. TERRAs are involved in telomere protection [17], regulation of telomerase activity [18], and heterochromatinization of subtelomeres [19]. TERRA promoters located on different subtelomeres that contain conserved repetitive DNA elements have been characterized [20]. In these promoters, a TSS, called TSSi hereafter, has been identified downstream of a CpG island (Figure 2). Methylation of this CpG island would negatively regulate TERRA expression [20]. RNA-seq data suggest that other TSSs are also located upstream in these subtelomere regions [21]. Thus, TERRA transcripts synthesized from the TSSs closer to the telomere, such as TSSi, overlap with TERRA transcripts that initiate from more distal TSSs. These transcripts do not contain unique sequences that allow quantification by classical RT-qPCR. 

Alu elements are primate-specific retroelements belonging to the class termed SINEs (short interspersed nuclear repetitive DNA elements). The human genome contains more than 1 million copies of Alu, which represent 11% of the genome. Alu elements contain a promoter for RNA polymerase III and are spread through the genome by retrotransposition [22]. The expression of Alu transcripts is regulated by epigenetic marks [23], flanking sequences [24] and transcription factors [25]. Because of these different layers of regulation, only a subset of Alu elements are expressed. Alu transcripts are necessary for the spreading of these mobile elements, but they have also been involved in gene regulation [26,27,28], epithelial-to-mesenchymal transition [29] and macular degeneration [30]. 

The specific quantification of Alu transcripts is also technically challenging. Because of the abundance of Alu elements within genes, a large number of pre-mRNA and mRNA transcribed by RNA polymerase II contain Alu sequences. These Alu elements included in RNA polymerase II transcripts are also amplify when a RT-qPCR is carried out with primers which hybridize to Alu sequence [22]. Moreover, the probes used to analyze Alu transcripts by northern blot hybridize also to the closely related 7SL RNA [22], which is much more abundant [31]. The primer extension approach enables one to perform a specific quantification of the Alu transcripts, but this method requires a large quantity of total RNA (around ten micrograms), which greatly limits the applications of this technique. 

Here, we demonstrate that the analysis of particular transcription start sites (AP-TSS) method that we have developed allows the relative quantification of TERRA from a specific TSSi and Alu transcripts from the RNA polymerase III promoter from a small amount of total RNA. AP-TSS is of interest to explore alternative TSS usage or the emerging function of Alu transcripts, and more generally their epigenetic regulation in the light of the development of epigenetic-targeted therapies. 

## 2. Materials and Methods 

### 2.1. Cell Culture 

Cell lines were purchased from ATCC. HT1080 (human fibrosarcoma) and HeLa (human cervix carcinoma) cell lines were cultured in DMEM, high glucose, GlutaMAX™ Supplement (Thermo Fisher, Illkirch, France), supplemented with 10% fetal calf serum and penicillin-streptomycin. For 5-azacytidine (5-aza-dC) treatment, cells were diluted to 25% confluence on day 0 in T-25 flasks and were treated, or not, with 10 µM 5-aza from day 1 to day 4. Cells were harvested on day 4. For α-amanitin treatment, HeLa cells plated in 6-well plate were treated or not with 50 µg/mL α-amanitin during 8 h. 

### 2.2. Extraction and Preparation of RNA

To perform this method, it is important to isolate high-quality RNA. We used phenol chloroform extraction to preserve RNA integrity. Cells grown in T-25 flasks (Techno Plastic Products, c Trassadigen, Switzerland) were centrifuged. Cell pellets were suspended in 1 mL of TRIzol™ Reagent (ThermoFisher), and 200 µL of chloroform was added. Samples were vortexed and centrifuged at 12,000× *g* for 15 min at 4 °C. The aqueous phase was collected, and RNA was precipitated with 1 volume of isopropanol. The pellets were washed with 70% ethanol, and RNA was dissolved in 50 µL H_2_O.

Since TERRA is present in small amounts in human telomerase-positive cell lines, it was essential to completely remove any DNA contamination using an extensive DNase I treatment. For this, 40 µg RNA (digestion was incomplete if a higher amount of RNA was used) were treated with 2 µL RNase-free DNase I (New England Biolabs, Evry, France ) in a 50 µL volume, including 5 µL 10× DNase I Reaction buffer, 1 µL RNase Inhibitor, Murine (New England Biolabs) for 30 min at 37 °C. RNA was precipitated with Lithium Chloride Precipitation Solution (Invitrogen), according to the manufacturer’s protocol, resuspended in 30 µL H_2_O, and quantified by UV spectrometry (Nanodrop, Thermo Fisher, Illkirch, France ).

### 2.3. Production of the Synthetic TERRA

Synthetic TERRA with a 5′ end identical to that of cellular TERRA initiated from TSSi was produced by in vitro transcription. PCR amplification of a subtelomeric amplicon located directly downstream of the TSSi was performed with TERRA-synth-R and TERRA-synth-F. TERRA-synth-R possesses a 5′ tail with the sequence of the T7 promoter, and TERRA-synth-F possesses a 5′ tail with five CCCTAA repeats. The PCR product was purified by agarose gel separation. In vitro transcription was performed with T7 RNA polymerase (New England Biolabs Evry, France). DNase I treatment was carried out to digest the PCR template, and purity of the RNA was monitored by agarose gel electrophoresis.

PCR with TERRA-synth-R and TERRA-synth-F was carried out with 50 ng of genomic DNA, 10 pmol of each primer, 0.4 nmol of dNTPs, 4 µL of Phusion High Fidelity buffer, and 0.2 µL of Phusion High Fidelity DNA Polymerase (New England Biolabs). The PCR was performed according to the following program: 1 cycle of denaturation at 98 °C for 30 s; 40 cycles of denaturation at 98 °C (10 s), annealing at 60 °C (30 s), and extension at 72 °C (30 s); and a final extension step at 72 °C (5 min). PCR products were run on a 1.5% agarose gel, and the band of the expected size was purified using the Gel Extraction Kit (EZNA, VWR, Fontenay-sous-Bois, France). In vitro transcription was performed with the HiScribe T7 High Yield RNA Synthesis Kit (New England Biolabs), according to the manufacturer’s protocol, by using the purified PCR product as a template. The DNase I digestion was performed as described above.

### 2.4. Reverse Transcription

To avoid incomplete reverse transcription due to RNA secondary structures, we used SuperScript III Reverse Transcriptase (Thermo Fisher). Due to high thermal stability, this enzyme can be used at 55 °C. TERRA was reverse transcribed using a primer complementary to the telomeric repeats (RP-TERRA). RP-TERRA and RP-Alu contain six phosphorothioate bonds in their 3′-terminal part, to block degradation of the cDNA during the RecJf digestion.

The reverse transcription reaction of TERRA extracted from cells was performed in 200-µL PCR tubes, in which 5 µg RNA, 20 pmol of RP-TERRA primer, 10 nmol of dNTPs, and H_2_O up to a final 13 µL volume were mixed. The samples were incubated in a PCR thermocycler at 65 °C for 5 min, transferred to ice, and 4 µL of 5X First Strand Buffer, 1 µL 0.1 M DTT, 1 µL RNase inhibitor, Murine (New England Biolabs), and 1 µL 200 U/µL SuperScript III RT or H_2_O for no-RT control were added. The samples were incubated in a PCR thermocycler at 55 °C for 60 min, followed by enzyme inactivation at 70 °C for 15 min. The reverse transcription of the synthetic TERRA was performed in the same conditions. For the reverse transcription of the Alu transcripts, the same protocol was performed using 1 µg of RNA and RP-Alu as primer.

### 2.5. Elongation of Specific cDNA

A template oligonucleotide (OT), with a 3′ region complementary to the 3′ end of the cDNA corresponding to TSS of interest, allows the 3′ elongation of only this cDNA (see Figure 2). In contrast, cDNA from an upstream TSS (TSS’) cannot be elongated, since template oligonucleotide hybridization with this cDNA does not form a 5′ overhang (i.e., a pseudo Y structure). As reverse transcriptase is known to add one to three cytosines at the 3′ end of cDNAs [32], the template oligonucleotide contains three guanines just upstream of the nucleotide, corresponding to the +1 position of the TSS of interest. The 5′ region of the template oligonucleotide corresponds to a unique sequence which is not found in the human genome. In addition, to prevent 3′ elongation of the template oligonucleotide, which would generate a false-positive signal, a dideoxycytidine was added to 3′ end. Importantly, in order to avoid removal of this dideoxycytidine, the cDNA elongation was performed using the modified Klenow fragment, lacking 3′→5′ exonuclease activity (Klenow exo-) (New England Biolabs).

To the TERRA cDNA samples was added 4 µL of a mix containing 1.25 nmol of dNTPs, 1.25 µmol of NaCl, 0.19 µmol of MgCl_2_, 0.5 µL of RNase A (20 mg/mL) (Invitrogen, Illkirch, France), and 0.5 pmol of OT oligonucleotide. These NaCl and MgCl_2_ concentrations are compatible with Klenow exo- and RecJf activities. Samples were incubated at 37 °C for 15 min to allow RNA digestion by RNase A. Hybridization of OT to the cDNA was carried out in a thermocycler by slow cooling (95 °C for 4 min, 57 °C for 5 min, 55 °C for 5 min, 50 °C for 5 min, 45 °C for 5 min, 40 °C for 5 min) after which samples were immediately transferred to ice. To perform elongation of the cDNA hybridized to the OT, 1 µL of Klenow exo- was added, and samples were incubated for 30 min at 37 °C. The specific elongation of Alu cDNA was performed in the same conditions, except that 5 pmol of template oligonucleotide (OT-Alu) was used because Alu transcripts are more abundant than TERRA.

### 2.6. Digestion of Primers Used for the Reverse Transcription and Template Oligonucleotides by RecJf

Primers used for the reverse transcription (RP-TERRA and RP-Alu) and template oligonucleotides (OT and OT-Alu) could interfere with subsequent qPCR. To eliminate these oligonucleotides, the 5′ exonuclease RecJf was used. Phosphorothioate bonds incorporated at the 3′-terminal region of RP-TERRA and RP-Alu oligonucleotides prevented the digestion of the cDNA by RecJf. Nonspecific products may be rarely generated during qPCR, due to the presence of residual undigested template oligonucleotides. In this case, a second digestion step by RecJf may be necessary. Samples were heated at 95 °C for 3 min to denature DNA. Samples were immediately transferred to ice, and 1 µL of RecJf (New England Biolabs) was added. Samples were incubated at 37 °C for 1 h and 40 min to digest oligonucleotides, and then at 75 °C for 20 min to inactivate RecJf.

### 2.7. Quantification of Elongated cDNA by qPCR and Data Analysis

Specific cDNA was then amplified by qPCR using a primer that anneals to the unique sequence added to the cDNA (P1 primer) and a primer that anneals to a sequence located downstream of the TSS of interest (P-TERRA or P-Alu primers).

Quantitative PCR was performed in an Mx3000P qPCR System (Agilent, Vénissieux, France), using Brilliant II SYBR^®^ Green qPCR Master Mix in a 96-well reaction plate (Agilent). For each qPCR sample, two technical replicates were carried out. Each reaction mix contained 5 pmol of each primer, and 1X Brilliant II SYBR^®^ Green qPCR Master Mix in a total volume of 25 µL. The plates were sealed with optically clear Strip Caps (Agilent). The qPCR were performed according to the following program: 1 cycle of denaturation at 95 °C for 15 min; 40 cycles of denaturation at 95 °C (10 s); annealing/extension at 60 °C (30 s); and dissociation for melting curve analysis. For each primer pair, a single peak was observed in dissociation curves, indicating amplification of a single amplicon (data not shown). Relative changes between samples were determined using the 2-ΔΔCt method with *GAPDH* mRNA, or 28S rRNA as reference transcripts, as described previously [33].

## 3. Results

### 3.1. Principle of the Method of Quantification of Transcript Based on Specific Elongation of the cDNA of Interest (AP-TSS)

We designed a new method to perform relative quantification by qPCR of the RNA transcribed from a unique TSS that we called AP-TSS. We used AP-TSS to the non-coding TERRA transcribed from a particular TSS present on several human subtelomeres (TSSi, Figure 2). Our approach is based on the addition of a sequence adaptor (Tag) at the 3′ end of the cDNA of interest, allowing further specific PCR amplification. Figure 2 schematically illustrates the principle of the assay for the quantification of TERRA from TSSi.

To check the sensitivity of the method, we used the telomerase-positive human cell line, in which TERRA level is low. Briefly, total RNA was extracted from cells and TERRA was reverse transcribed, using a primer complementary to the telomeric UUAGGG repeats (RP-TERRA). The 3′ end of the cDNA corresponding to the targeted TSS was hybridized to the 3′ region of a template oligonucleotide (OT), containing a Tag sequence at the 5′ end. Oligonucleotide sequences are listed in Table 1. The 5′ region of OT is a sequence not found in the human genome. In this template oligonucleotide, three guanines are present just upstream of the nucleotide corresponding to the +1 position of the TSSi, because reverse transcriptase is known to add one to three cytosines at the 3′ end of newly synthesized cDNA [32]. In addition, there is a dideoxycytidine at the 3′ end of OT, in order to prevent further elongation by polymerases (Table 1). Hybridization of OT to the TSSi cDNA generates a 5′ overhang, allowing the 3′ recessed cDNA to be elongated by a polymerase without exonuclease activity to avoid the loss of the dideoxycytidine; this incorporates the Tag sequence at the 3′ end of the TSSi cDNA. Then, a qPCR is carried out with a primer complementary to the tag sequence and a primer complementary to a sequence located downstream of the TSSi, which enables specific amplification of the TSSi cDNA in the presence of cDNAs encoding other TERRA isoforms. In order to eliminate oligonucleotides OT and RP-TERRA, which could interfere with the qPCR, a digestion with a 5′-3′ exonuclease, RecJf, was performed before the qPCR step. To avoid digestion of the cDNA by RecJf, six phosphorothioate bonds were included at the 3′ terminus of the RP-TERRA oligonucleotide (Table 1). Reverse transcription, elongation, and digestion steps were performed in the same tube, avoiding DNA loss. The cDNA of interest was specifically amplified in qPCR assay using primer P1, complementary to the Tag sequence, and primer P-TERRA, which is complementary to a sequence located downstream. Oligonucleotide OT used for cDNA elongation and primers P1 and P-TERRA used for qPCR have been designed for the quantification of the subtelomeric TSSi transcript synthesized from chromosomes Xq, Yq, 15q, and 9p.

### 3.2. Validation of the Method on a Synthetic TERRA

To validate the method and demonstrate that it enables a relative quantification, a sufficient amount of transcript of interest was necessary. Given that the TERRA level is very low in telomerase-positive cell lines, we used a synthetic TERRA. This synthetic TERRA was produced by in vitro transcription of a PCR product obtained, by amplifying the subtelomeric sequence located directly downstream of the TSSi (Figure S1). Thus, the 252 nucleotides long synthetic TERRA has the same sequence as the 5′ part of the cellular TERRA from the TSSi.

**Figure 3:**
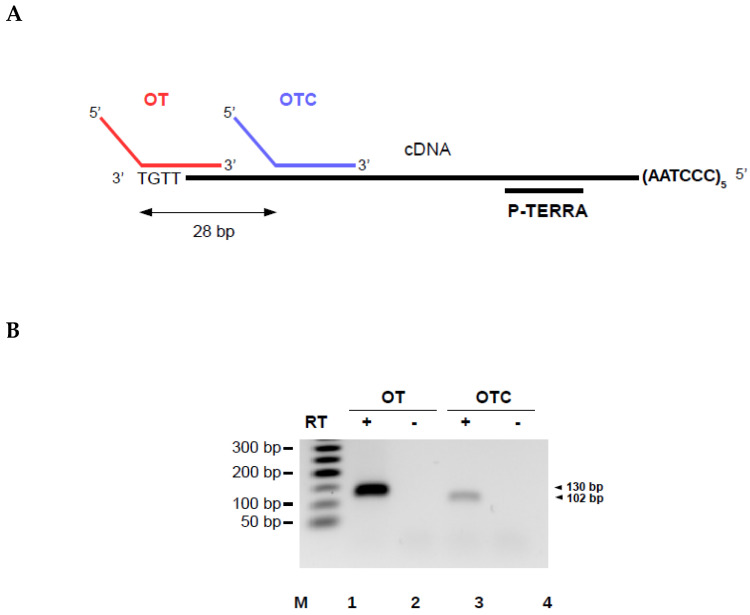

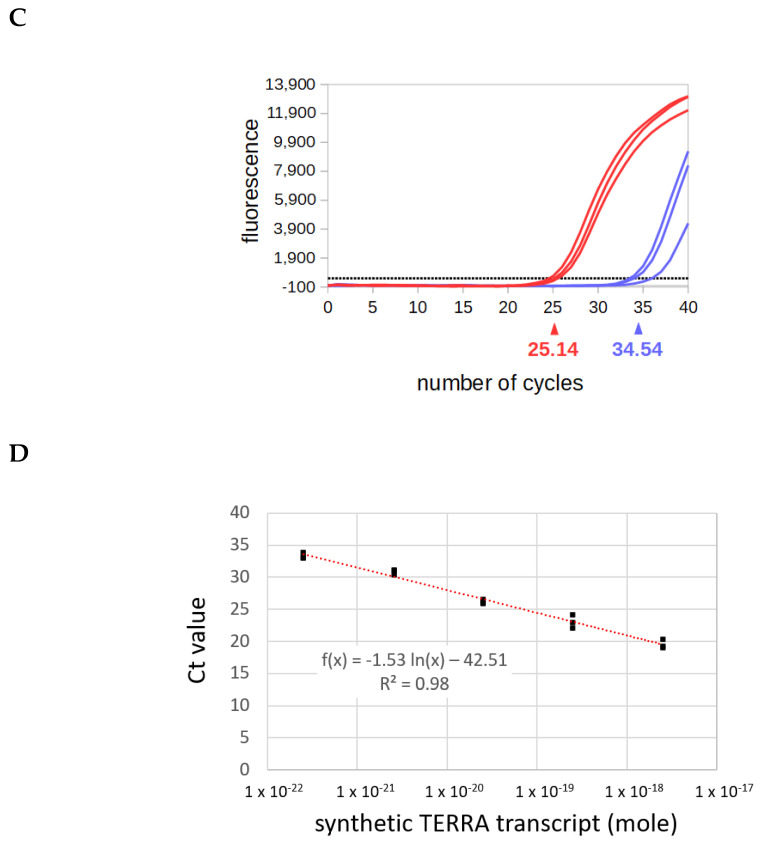
Validation of the AP-TSS method. (**A**) Schematic illustration showing locations of OT and OTC bound to the cDNA obtained by reverse transcription of synthetic TERRA with RP-TERRA. (**B**) Synthetic TERRA was reverse transcribed (RT) (lanes 1 and 3) or not (lanes 2 and 4), using RP-TERRA as a primer. Elongation was carried out using OT or OTC. Elongation products were amplified by PCR using P1 and P-TERRA, and PCR products (130 bp and 102 bp, arrowhead on the right) were run on a 2% EtBr-agarose gel. Lane M is the 50-bp DNA ladder (New England BioLabs). (**C**) Amplification plot of a qPCR assay after elongation step performed with OT or OTC. 2.10^-18^ moles of synthetic TERRA was reverse transcribed using RP-TERRA as primer, the elongation of the cDNA was performed by OT or OTC and 1/15 of samples was used for the qPCR with P-TERRA and P1 primers. Graph shows the fluorescence as a function of the number of PCR cycles for three samples elongated with OT (red curves) or OTC (blue curves). The black dashed line corresponds to the threshold. The average Ct value for samples elongated with OT and OTC are indicated in red and blue, respectively. (**D**) Five dilutions of synthetic TERRA transcript were reverse transcribed using RP-TERRA as primer, the elongation of the cDNA was performed by OT and 1/70 of each sample was used for the qPCR with P-TERRA and P1 primers. Graph shows the triplicates of Ct values as a function of the logarithm of the corresponding synthetic TERRA quantity (in moles). The equation and R^2^ of the regression line (in red) are indicated.

To validate the AP-TSS method, synthetic TERRA was reverse transcribed using RP-TERRA and elongation was performed with the oligonucleotide OT (Figure 3A). The expected qPCR product of 130 base pairs (bp) was obtained in the presence (lane 1) but not in the absence (lane 2) of reverse transcriptase (Figure 3B).

The specificity of our approach was explored. No qPCR product should be obtained by AP-TSS when the 3′ region of the template oligonucleotide is complementary to an internal sequence of the cDNA instead of the 3′ end. To experimentally verify this, a template oligonucleotide control (OTC) was designed. The 5′ half of the oligonucleotide is identical to OT, but the 3′ region is complementary to a sequence located 28 bp downstream of the 3′ end of the cDNA (Figure 3A). Unexpectedly, a weak qPCR product of 102 bp was obtained with OTC (Figure 3B, lane 3). Sequencing of this product showed that it corresponds to the subtelomeric sequence located between OTC and P-TERRA. Nevertheless, the amplification plot of the qPCR showed that the threshold was crossed by the fluorescent signal for a significantly higher number of cycles with OTC than with OT (Figure 3C). Thus, the average cycle threshold (Ct) value obtained by qPCR was 25.14 +/− 0.48 with OT and 34.54 +/− 1.29 with OTC.

Two possible events could explain the weak 102-bp product obtained with OTC. First, OTC may have hybridized to rare molecules of cDNA with a 3′ end complementary to OTC; this could have been the case if the RNA was partially degraded, or if reverse transcription was not complete. Second, OTC might be elongated despite blocking of the 3′ terminus by dideoxycytidine. This could have happened if the 3′-termini of all OTC oligonucleotides were not modified, which is unlikely, as template oligonucleotides were purified by PAGE (polyacrylamide gel electrophoresis), or if the 3′ modification was lost. If the template oligonucleotide was elongated, it would be amplified by qPCR and generate the 102-bp product. To be amplified by qPCR, the elongated template oligonucleotide must, in addition, have escaped RecJf digestion; however, this 5′ exonuclease digests the large majority of the template oligonucleotides (Figure S2).

To demonstrate that the method enables a relative quantification, the analysis was performed on a serial dilution of synthetic the TERRA transcript. The efficiency of PCR in a qPCR assay must be precisely determined and be within an acceptable range for quantitative analysis. The most reliable and robust method for determination of PCR efficiency is by means of a standard curve constructed by serial dilution of a concentrated stock solution of DNA template. Amplification efficiency calculated from the slope of the standard curve should be between 90% and 110% (corresponding to a slope between −1.558 and −1.348), and the R^2^ must be higher than 0.98. The synthetic TERRA produced by in vitro transcription was serially diluted, and each RNA sample was subjected to quantification using our method. After reverse transcription of each RNA sample using RP-TERRA as primer, the cDNA was elongated with the template oligonucleotide OT. After RecJf-digestion of the sample, a qPCR with P-TERRA and P1 primers was carried out with 1/70 of each sample. The Ct values of triplicate samples were plotted as a function of the logarithm of the corresponding synthetic TERRA quantity (Figure 3D). The slope of the regression line is −1.531 (which corresponds to an efficiency of 92.2%), and the R^2^ is equal to 0.982. This result demonstrates that AP-TSS enables the relative quantification of TSSi TERRA transcripts. Noteworthy, AP-TSS enables a relative quantification when the initial quantity of synthetic TERRA in the sample is 2.5 × 10^−22^ mol, which corresponds to approximatively 150 molecules.

### 3.3. Application of AP-TSS to Compare the Levels of Transcripts from a Particular TERRA TSS Under Different Conditions

**Figure 4:**
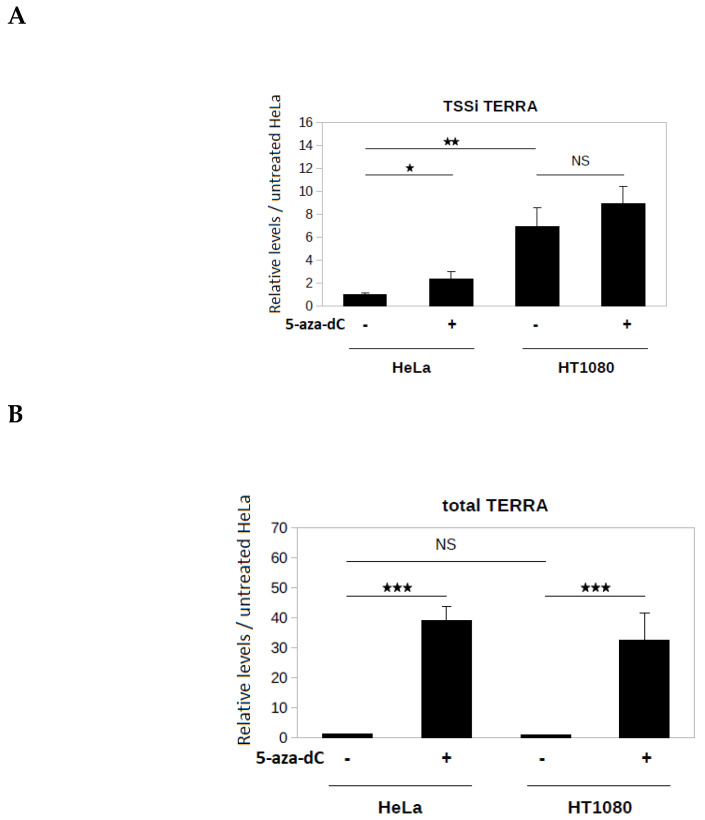
Application of AP-TSS to TERRA extracted from HeLa and HT1080 cells. (**A**) Measure of the level of TSSi TERRA by AP-TSS. RNA from HeLa and HT1080 cells untreated or treated with 5-aza-dC was reverse transcribed using RP-TERRA as a primer. Elongation was carried out using OT. TERRA produced from the TSSi located on subtelomeres Xq, Yq, 15q, and 9p was specifically quantified by qPCR using P1 and P-TERRA primers; levels were normalized to *GAPDH* RNA, and all values were compared to untreated HeLa sample. (**B**) Measure of the level of total TERRA by classical qPCR. RNA from HeLa and HT1080 cells untreated or treated with 5-aza-dC was reverse transcribed using RP-TERRA as a primer. TERRA from subtelomeres Xq, Yq, 15q, and 9p (total TERRA) was quantified by qPCR using TR and TF primers; levels were normalized to *GAPDH* RNA, and all values were compared to untreated HeLa sample. The bars represent the average values from three biological and two technical replicates for each sample. Error bars represent the standard deviations. P-values were calculated by paired two-tailed Student t-test (*n* = 3). NS, not significant, * *p* <0.05, ** *p* <0.01, *** *p* < 0.001.

Subtelomeres Xq, Yq, 15q, and 9p belong to a subtelomere family possessing a CpG island located upstream of the TSSi. This CpG island is strongly methylated in telomerase-positive human cell lines, and this methylation has been shown to negatively regulate the level of TERRA from these subtelomeres [20]. To investigate the influence of CpG island methylation on transcription from TSSi, HeLa and HT1080 cells were treated with the DNA methyltransferase (DNMT) inhibitor 5-aza-2′-deoxycytidine (5-aza-dC). It has been shown that this treatment is associated with a demethylation of the subtelomeric CpG island [20]. Application of AP-TSS to TSSi showed that a three-day treatment with 5-aza-dC (10 µM) induced a significant 2.3-fold increase in TSSi TERRA level in HeLa cells, but did not significantly alter TSSi TERRA levels in HT1080 cells (Figure 4A). In the absence of 5-aza-dC treatment, the TSSi TERRA level was 6.9-fold higher in HT1080 cells than in HeLa cells (Figure 4A).

Total TERRA produced from subtelomeres Xq, Yq, 15q, and 9p was quantified by qPCR using primers TF and TR located downstream of the TSSi (Figure 2). Notably, 5-aza-dC treatment induced a significant increase of the total TERRA level compared to untreated control in both cell lines (37-fold increase in HeLa cells, 43-fold increase in HT1080 cells) (Figure 4B). Of note, in the absence of 5-aza-dC treatment, no significant difference in the total TERRA level was observed between the two cell lines (Figure 4B), whereas the TSSi TERRA level was significantly higher in HT1080 cells than in HeLa cells (Figure 4A). This suggests that other subtelomeric TSSs are activated, as previously reported [21]. The strong increase of total TERRA induced by 5-aza-dC treatment in the two cell lines, compared to the modest change (HeLa) or absence of change (HT1080) in the TSSi TERRA level (Figure 4A), indicates that the high level of TERRA in 5-aza-dC treated cells was mainly due to TERRA transcription from subtelomeric TSSs other than TSSi.

### 3.4. Application of the Method to the Alu Transcripts

**Figure 5:**
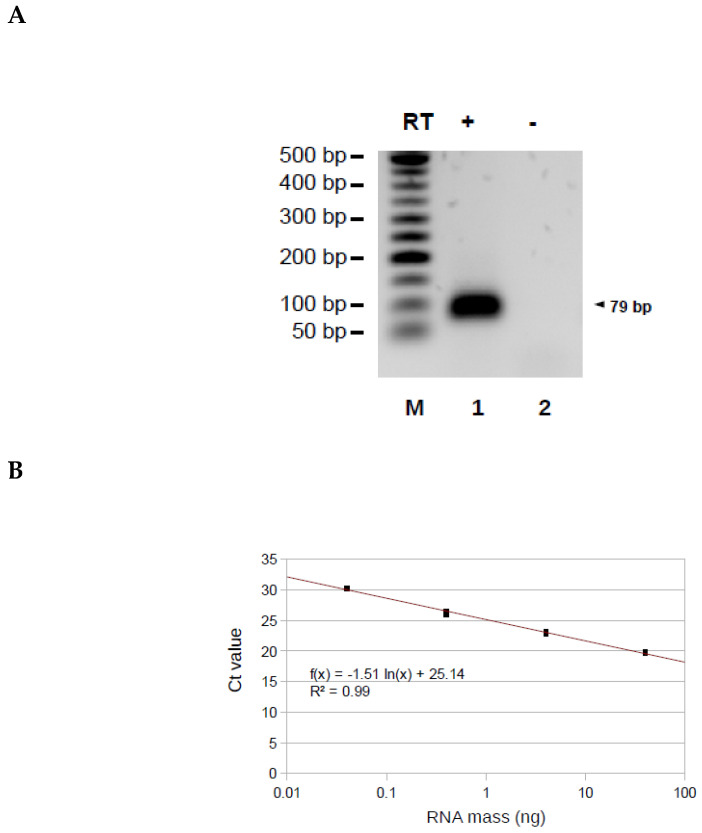

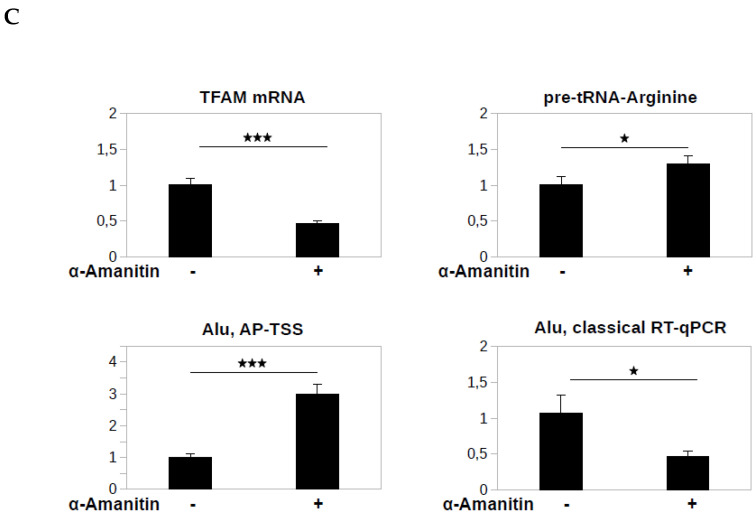
Application of AP-TSS to Alu transcripts. (**A**) RNA extracted from HeLa cells was reverse transcribed (RT) (lane 1) or not (lane 2), using RP-Alu as a primer. Elongation was carried out using OT-Alu. Elongation products were amplified by PCR using P1 and P-Alu, and PCR products (79 bp, arrowhead on the right) were run on a 1.5% EtBr-agarose gel. Lane M is the 50-bp DNA ladder (New England BioLabs). (**B**) Four dilutions of total RNA were reverse transcribed using RP-Alu as primer, the elongation of the cDNA was performed by OT-Alu and 1/50 of each sample was used for the qPCR with P-Alu and P1 primers. Graph shows the triplicates of Ct values as a function of the logarithm of the corresponding RNA mass (in ng). The equation and R^2^ of the regression line (in red) are indicated. (**C**) HeLa cells were untreated or treated for eight hours with 50 µg/mL α-amanitin and RT-qPCR with primers hybridizing to pre-tRNA-Arginine, *TFAM* mRNA or Alu sequence (Alu, classical RT-qPCR) were performed. RNA was reverse transcribed using RP-Alu as a primer and elongation was carried out using OT-Alu, followed by qPCR using P1 and P-Alu (Alu, AP-TSS). All the qPCR results were normalized to 28S rRNA, a stable rRNA transcribed by RNA polymerase I, and all values were compared to untreated HeLa sample. The bars represent the average values from three biological and two technical replicates for each sample. Error bars represent the standard deviations. *P*-values were calculated by paired two-tailed Student t-test (*n* = 3). NS, not significant, * *p* < 0.05, ** *p* < 0.01, *** *p* < 0.001.

To determine if our approach can be used on other transcripts from particular TSSs other than TERRA, we applied it on the Alu transcripts in total RNA extracted from HeLa cells. The reverse transcription used a primer RP-Alu hybridizing to position 217-238 Alu PV consensus [34] and the elongation step was carried out using the primer OT-Alu, whose 3′ part is identical to position 1-20 Alu PV consensus. The expected qPCR product of 79 base pairs (bp) was obtained in the presence (lane 1) but not in the absence (lane 2) of reverse transcriptase (Figure 5A). To demonstrate that AP-TSS enables the relative quantification of Alu transcripts, the method was applied to four samples obtained by serial dilution of total RNA extracted from HeLa cells. The Ct values were plotted as a function of the logarithm of the corresponding total RNA mass and showed an efficient linear regression (R^2^ = 0.994), corresponding to a qPCR efficiency of 93.6% (Figure 5B). This result demonstrates that AP-TSS enables to perform a relative quantification of Alu transcripts, even when a tiny amount of total RNA (0.04ng) is used. It has been estimated that the quantity of Alu transcripts in a HeLa cell is approximatively 100 molecules [35]. As the total RNA mass in a cell is around 10pg, a total RNA mass of 0.04ng corresponds approximatively to 400 molecules of Alu transcripts.

To demonstrate the specificity of AP-TSS, it was necessary to show that the qPCR signal is generated by the Alu transcripts and not by the RNA polymerase II transcribed RNAs containing Alu elements. Liu et al. have reported that the specific inhibition of RNA pol II by α-amanitin induces an increase of the Alu transcripts level by a mechanism which is still poorly understood [31]. This effect was obtained by primer extension assay, which enables the specific quantification of the Alu transcripts [22]. We reasoned that if our method is specific to RNA Pol III-dependent Alu transcripts, the α-amanitin treatment would increase the qPCR signal. On the other hand, if the signal is mainly generated by RNA polymerase II transcribed RNAs containing Alu elements, the α-amanitin treatment would decrease the qPCR signal. We extracted total RNA from HeLa cells treated, or not, with 50 µg/mL α-amanitin for 8 h. This treatment induced a 2.2-fold decrease of the level of mRNA of TFAM (Transcription Factor A, Mitochondrial) transcribed by RNA polymerase II, but did not decrease the level of pre-tRNA-Arginine transcribed by RNA polymerase III (Figure 5C), that were used as controls to validate our experimental conditions to specifically inhibit RNA Polymerase II with α-amanitin. For ALU, the α-amanitin treatment induced a 3-fold increase of the signal generated by using AP-TSS, thus validating the specificity of our approach. On the other hand, the α-amanitin treatment induced a 2.2 fold decrease of the signal generated by classical RT-qPCR with primers ALU-F and ALU-R, which hybridize to position 82-103 and 238-222 Alu PV consensus respectively, suggesting that the signal obtained by RT-qPCR is, at least partially, generated by the amplification of Alu elements included in RNA polymerase II transcripts.

## 4. Discussion

Here, we describe an accurate and sensitive method for relative quantification of transcripts produced from a given TSS. This method, called AP-TSS, is based on the specific 3′ elongation of the cDNA produced from a single TSS, using a template oligonucleotide designed to be complementary to the 3′ end of the targeted cDNA, followed by its quantification by qPCR. Many genes have multiple transcription start sites. In some cases, the transcript isoforms produced do not have unique sequence, as the whole sequence of the shorter isoform is contained in that of the longest isoform. In this case, an RT-qPCR assay designed to detect the shorter transcript also amplifies the longer isoform. Two types of approaches have been used to detect and estimate the amounts of transcript isoforms. The first ones include techniques such as CAGE, RACE, and RACE-seq that involve addition of an adaptor at the 3′ end of the cDNA, followed by PCR amplification [12,16,36]. These methods are well-suited for the discovery and detection of isoforms, but are semi-quantitative because of the bias introduced during addition of the adaptor (by ligation or template switching) [37]. Furthermore, these techniques are expensive and time consuming. The second ones are based on the comparison between the number of transcripts possessing a sequence located downstream of the TSS to the number of transcripts possessing a sequence located upstream of the TSS. This can be done by RNA-seq [38], which is imprecise for weakly expressed transcripts [39], or by RT-qPCR, which requires a comparison of results obtained with two different primer sets [40,41]. These methods cannot be used to quantify transcript isoforms present in small numbers compared to numbers of transcript isoform produced from the upstream TSS. Indeed, in both approaches, the difference between the signals obtained from the upstream and from the downstream of the TSS are negligible compared to each of these signals.

Here, we describe a straightforward methodology that allows the relative quantification of transcript produced from a previously identified TSS. The addition of a sequence adaptor at the 3′ end of the cDNA of interest is based on the hybridization of an oligonucleotide, with this cDNA followed by elongation. Compared to RACE and CAGE methods, our approach has the advantage that only the cDNA of interest is elongated, enabling its specific PCR amplification. Moreover, the elongation step does not introduce the same bias as in a ligation step [37]. Nevertheless, contrary to RACE and CAGE, AP-TSS does not enable the identification of new TSSs. We demonstrated the validity of our approach on a synthetic RNA TERRA and on Alu transcripts. AP-TSS was also applied to TERRA from HeLa or HT1080 cells after treatment with 5-aza-dC. Our data provide evidence that this method can be applied to perform relative quantification of a transcript from a particular TSS, even expressed at a low level.

First, to validate the AP-TSS method, the synthetic TERRA produced by in vitro transcription was reverse transcribed and a template oligonucleotide complementary to the 3′ end of the cDNA (OT) was used. A template oligonucleotide complementary to a sequence located 28-bp downstream of the 3′ end of the cDNA (OTC) was used as control. A unique qPCR product with the expected sequence was obtained with OT, with an average Ct value of 25.14 +/− 0.48. However, with OTC a qPCR product was also detected with a high average Ct value of 34.54 +/− 1.29; this product was 28 bp shorter than the one obtained with OT.

Two possible events could have occurred to cause the production of this 102-bp qPCR product using OTC as template oligonucleotide. First, the control template oligonucleotide could have hybridized to the 3′ end of a truncated cDNA; this could have been the case if the RNA were partially degraded, or if reverse transcription was not complete. Second, elongation of the control template oligonucleotide despite blocking of the 3′ terminus by dideoxycytidine, followed by amplification, could have resulted in these products. Indeed, although Figure S2 shows that the large majority of the template oligonucleotides are digested by RecJf, it is likely that a tiny amount undetectable by ethidium bromide staining escape to the digestion. In anyway, this suggests that a signal can be generated by transcripts from upstream TSSs. Nevertheless, a difference between average Ct values of 9.4 cycles was observed with OT and OTC. Although the Ct values are not directly comparable, as the amplicons are not the same, we can estimate that the specific signal is around 700 (~2^9.4^) fold higher than nonspecific signal. Thus, AP-TSS is substantially more sensitive than other methods using qPCR or RNA seq to compare the level of transcripts on both sides of the TSS of interest [38,40,41]. Nonetheless, because of the possibility that a transcript from upstream TSS may generate a signal, we recommend to use AP-TSS to compare expression from a particular TSS between different conditions, rather than to determine if a particular TSS is active or not in a given context. Because of their higher specificity, CAGE and RACE methods are more suitable to determine if a TSS is expressed or not. Indeed, one feature of the 5′ RACE and CAGE methods is that they detect only transcripts with capped 5′ ends. Thus, uncapped transcripts resulting from post-transcriptional degradation do not generate signal. Nevertheless, it should be noted that the specific detection of capped transcripts does not completely eliminate nonspecific signals. Indeed, a cytoplasmic complex adding caps to RNA after processing has been identified [42], and it has been shown that some CAGE tags result from cytoplasmic recapping events [43]. Thus, even when 5′ RACE and CAGE methods are used to evaluate expression from a TSS of interest, transcripts from upstream TSSs may generate positive results.

To prove the validity of our approach, we used an endogenous transcript, the SINEs element ALU that is transcribed by RNA polymerase III. We applied it to the TSS of the RNA polymerase III promoter located in the Alu sequences. We took advantage of the opposite effect of the α-amanitin treatment on the level of RNA polymerase III transcribed Alu RNAs and on the level of RNA polymerase II transcribed RNAs that contain Alu sequences [31], to show that the signal generated by AP-TSS is specific to the Alu transcripts, in contrast to classical RT-qPCR. To demonstrate that this technique allowed the relative quantitation of transcripts of interest, we analyzed serial dilutions of synthetic TERRA or from cellular RNA for Alu transcripts. The parameters of the resulting standard curves were in accordance with the criteria of validity of quantitative PCR. A similar result was obtained by applying the method to the canonical TSS of the GAPDH gene (data not shown). Altogether, these results demonstrate the robustness of AP-TSS to perform a relative quantification. Notably, these standard curves show that AP-TSS enables relative quantification, even for a tiny number of transcripts of interest (around 100 molecules).

Our method enables the comparison of the expression of a transcript isoform from a given TSS under different conditions (e.g., treatment with drug, different cell types, development stage, or cell-cycle stage). An important application will be the study of how epigenetic modifications influence the use of specific TSSs. It is less appropriate to compare the expression levels of transcripts produced from two different TSSs, because this would require a comparison of the PCR results obtained with two different amplicons. Nevertheless, classical RT-qPCR also has this limitation.

As a proof of principle, AP-TSS was used to analyze TERRA levels from the TSSi in two cell lines (HT1080 and HeLa). Although TERRA expression was previously reported to be negatively regulated by cytosine methylation in its promoter [20], no information was available about the regulation of TERRA transcript isoforms. Using our approach, we investigated the effect of DNMT inhibition by 5-aza-dC treatment on transcription from TSSi and on total TERRA levels in HT1080 and HeLa cell lines. The 5-aza-dC treatment inhibitor did not alter TSSi transcript levels in HT1080 cells and induced a modest 2.3-fold increase in HeLa cells, whereas TERRA expression from subtelomeres Xq, Yq, 15q, and 9p was increased by 39- and 24-fold in HT1080 and HeLa cells, respectively. This result indicates that the strong up-regulation of TERRA induced by 5-aza-dC treatment was mainly due to TERRA transcription from subtelomeric TSSs other than TSSi. In line with these results, previous studies have shown that the genome demethylation by 5-aza-dC treatment has a profound effect on TSS usage [5,6,36].

This method provides an efficient tool for the study of the regulation of alternative TSS use. This is of particular interest, as for certain genes, different TSSs are used over the course of normal development [44] and in diseased compared to normal tissue [2]. Regulation of transcription start site selection is poorly understood. In particular, understanding the epigenetic regulation of alternative TSS usage is of importance for the development of epigenetic-targeted therapies [45]. In addition to TSS switching, another process that contributes to transcript diversity is post-transcriptional RNA cleavage [46]. Uncleaved and cleaved forms of transcripts produced, for example from the *D4Z4* gene [47], can be present simultaneously. In this case, the cleaved form cannot be quantified by classical RT-qPCR. AP-TSS could be used to quantify the transcript isoforms produced by post-transcriptional cleavage, enabling the study of the regulation of this process.

## 5. Conclusions

In this work, we describe a method to perform relative quantification of a transcript from a particular TSS. Our approach, called AP-TSS, is based on the specific 3′ elongation of the cDNA of interest, followed by relative quantification by qPCR. AP-TSS can be used to compare the expression between different conditions of an isoform transcript which cannot be specifically quantified by classical RT-qPCR, as its sequence is contained in longer isoforms of the gene. This is of special interest because usage of alternative TSS participates in gene regulation and proteome diversity. As a proof a principle, we applied AP-TSS to a previously identified TSS of the telomeric transcript TERRA, and to the Alu transcripts. This showed that the strong increase of TERRA level induced by 5-aza-dC treatment was mainly due to an increase of the expression from others subtelomeric TSSs.

## Figures and Tables

**Table 1:** Oligonucleotide sequences.

Oligonucleotide	Sequence (5′-3′)
RP- telomeric repeat-containing RNA (TERRA)	CCCTAACCCTAACCCTAACCCT*A*A*C*C*C*TAA
OT	GCACTCGGTGAGTCGTACTACGGGACAACTCGGGGCGCATCAAddC
OTC	GCACTCGGTGAGTCGTACTACGGGAAAATGTTTCCCGGTTGCAGCddC
P1	GCACTCGGTGAGTCGTACTACG
P-TERRA	CTTTCCCGTTTTCCGCACTG
TF	GCAGCCATGAATAATCAAGGT
TR	TTCCGCACTGAACCGCTCTAA
GAPDH-R	GAAGGTGAAGGTCGGAGTCAAC
GAPDH-F	CAGAGTTAAAAGCAGCCCTGGT
TERRA-synt-R	CTATAATACGACTCACTATAGACAACTCGGGGCGCATCA
TERRA-synt-F	CCCTAACCCTAACCCTAACCCTAACCCTAATTGTGTGCATTAGGAATGCTG
RP-Alu	GCGATCTCGGCTC*A*C*T*G*C*AAG
OT-Alu	GCACTCGGTGAGTCGTACTACGGGGGCCGGGCGCGGTGGCTCACddC
P-Alu	CCGCCTCGGCCTCCCAAAGT
Alu-F	ACCATCCCGGCTAAAACGGTGA
Alu-R	GCGATCTCGGCTCACTG
TFAM-F	GTGGGAGCTTCTCACTCTGG
TFAM-R	TAGGGCTTTTTCTCCTGCAA
pre-tRNA-Arg-F	GGCTCTGTGGCGCAATGGATA
pre-tRNA-Arg-R	TTCGAACCCACAACCTTTGAATTGCTC
ARN-28S-F	TGTTAGGACCCGAAAGATGG
ARN-28S-R	TCGGAGGGAACCAGCTACTA

*: phosphorothioate bond. ddC: dideoxycytidine.

## Supplementary Materials

The following are available online at https://www.mdpi.com/2218-273X/10/6/827/s1, Figure S1: Schematic illustration showing the in vitro transcription of the synthetic TERRA, Figure S2: Digestion of the template oligonucleotide OT by RecJf.

## Abbreviations

5-aza-dC	5-aza-2′-deoxycytidine
CAGE	cap analysis of gene expression
Ct	cycle threshold
DNMT	DNA methyltransferase
EtBr	ethidium bromide
GAPDH	glyceraldehyde-3-phosphate dehydrogenase
OT	oligonucleotide template
OTC	oligonucleotide template control
PAGE	polyacrylamide gel electrophoresis
RACE	rapid amplification of cDNA ends
RNA-seq	RNA sequencing
RT-qPCR	reverse transcription quantitative polymerase chain reaction
SINE	short interspersed nuclear elements
TERRA	telomeric repeat-containing RNA
TSS	transcription start site
TSSi	transcription start site of interest
UTR	untranslated region
