# Supplementary material for: AP-TSS: A New Method for the Analysis of RNA Expression from Particular and Challenging Transcription Start Sites
Source: Biomolecules. 2020 May 28;10(6):827. doi: 10.3390/biom10060827 (PMC7355800; doi:10.3390/biom10060827)
Supplement: Supplementary file 1 [file biomolecules-10-00827-s001.pdf]

# AP-TSS: a new method for the analysis of RNA expression from particular and challenging transcription start sites

Le Berre G., *et al.*

## Supplementary Material

Figure S1. Schematic illustration showing the in vitro transcription of the synthetic TERRA
Figure S2. Digestion of the template oligonucleotide OT by RecJf

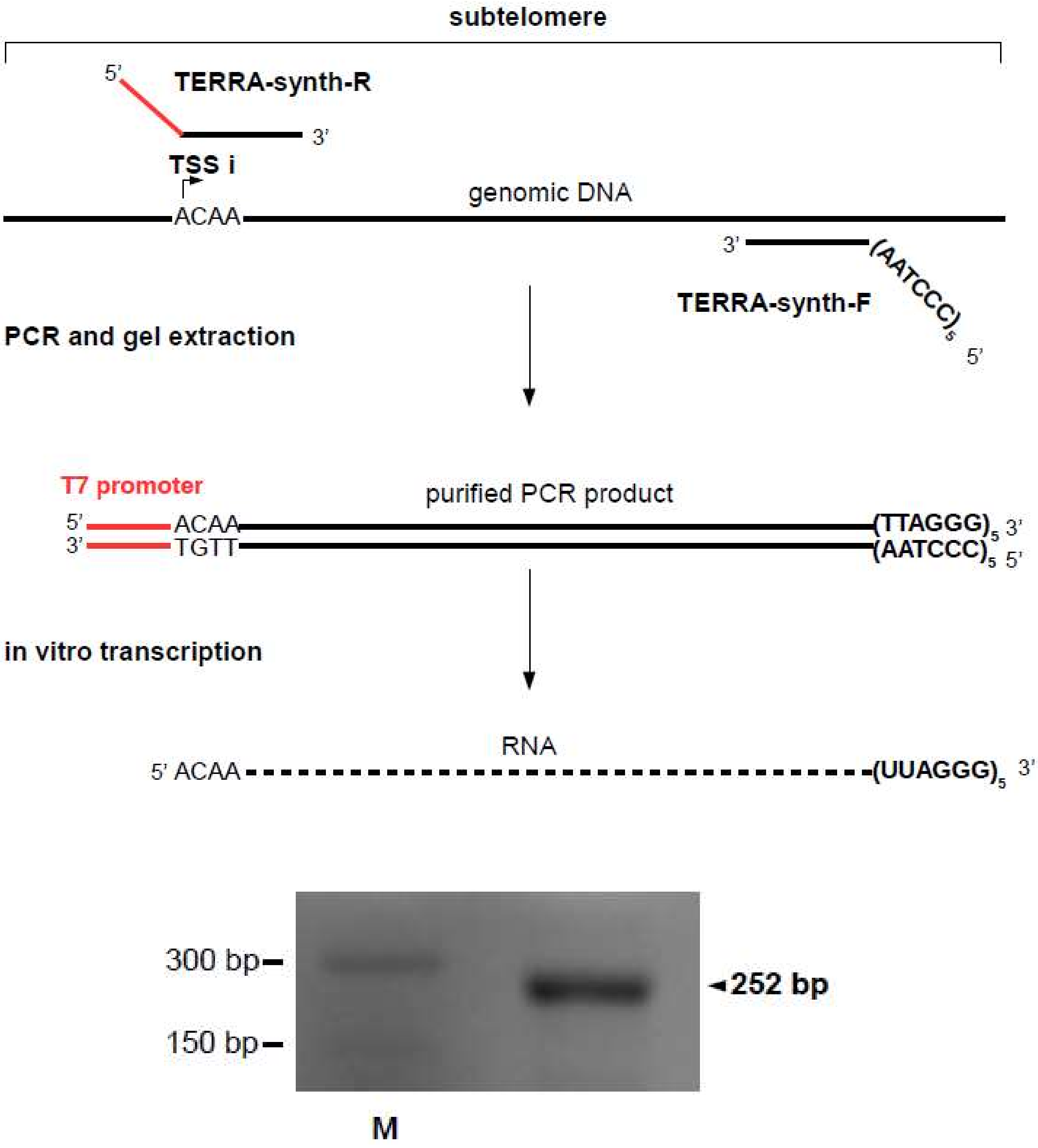


**Figure S1.** Schematic illustration showing the in vitro transcription of the synthetic TERRA. PCR with TERRA-synth-R and TERRA-synth-F was performed from genomic DNA of HT1080 cells. TERRA-synth-R is complementary to a sequence directly downstream of the TSSi and possesses a 5′ tail containing the sequence of the T7 promoter (red). TERRA-synth-F is complementary to a sequence located 200 bp downstream of the TSSi and possesses a 5′ tail with five CCCTAA repeats. The PCR product of the expected size was purified from a gel. Transcription in vitro was carried out with T7 RNA polymerase. The image of the gel shows the sample from in vitro transcription run on a 2% EtBr-agarose gel. A transcript of the expected size (252 bp) was obtained (indicated by arrow).

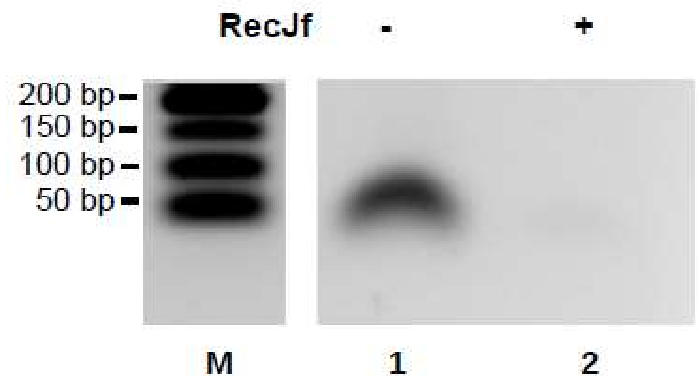


**Figure S2.** Digestion of the template oligonucleotide OT by RecJf. OT (10 pmol) was incubated at 37 °C for 1 h and 40 min in 20 μL of NEBuffer 2 in the absence (lane 1) of in the presence (lane 2) of RecJf. Lane M is the 50-bp DNA ladder (New England BioLabs). Samples were incubated at 75 °C to inactivate RecJf and were run on a 2% EtBr-agarose gel.
